# The progression of traumatic Stanford type A acute aortic dissection

**DOI:** 10.1002/ccr3.7479

**Published:** 2023-06-13

**Authors:** Hironobu Nishiori, Hisanori Fujita, Seiichi Yamaguchi

**Affiliations:** ^1^ Department of Cardiovascular Surgery Chiba Emergency Medical Center Chiba Japan

**Keywords:** aortic dissection, blunt injury, cardiopulmonary bypass

## Abstract

**Key Clinical Message:**

Cardiopulmonary bypass for trauma patients carries the risk of bleeding from injured organs, while traumatic aortic dissection can progress rapidly. It is sometimes difficult to determine the optimal time for aortic repair in trauma patients.

**Abstract:**

An 85‐year‐old woman was diagnosed with traumatic ascending aortic dissection, right clavicle and left first rib fracture, and abdominal contusions after a vehicle accident. After admission, the aortic dissection progressed, and emergent surgery was performed. Although the risk of hemorrhagic complications needs to be evaluated, prompt aortic repair is required.

## Graphical abstract

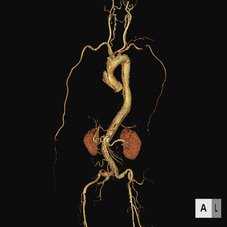

## 1 CASE

**FIGURE 1:**
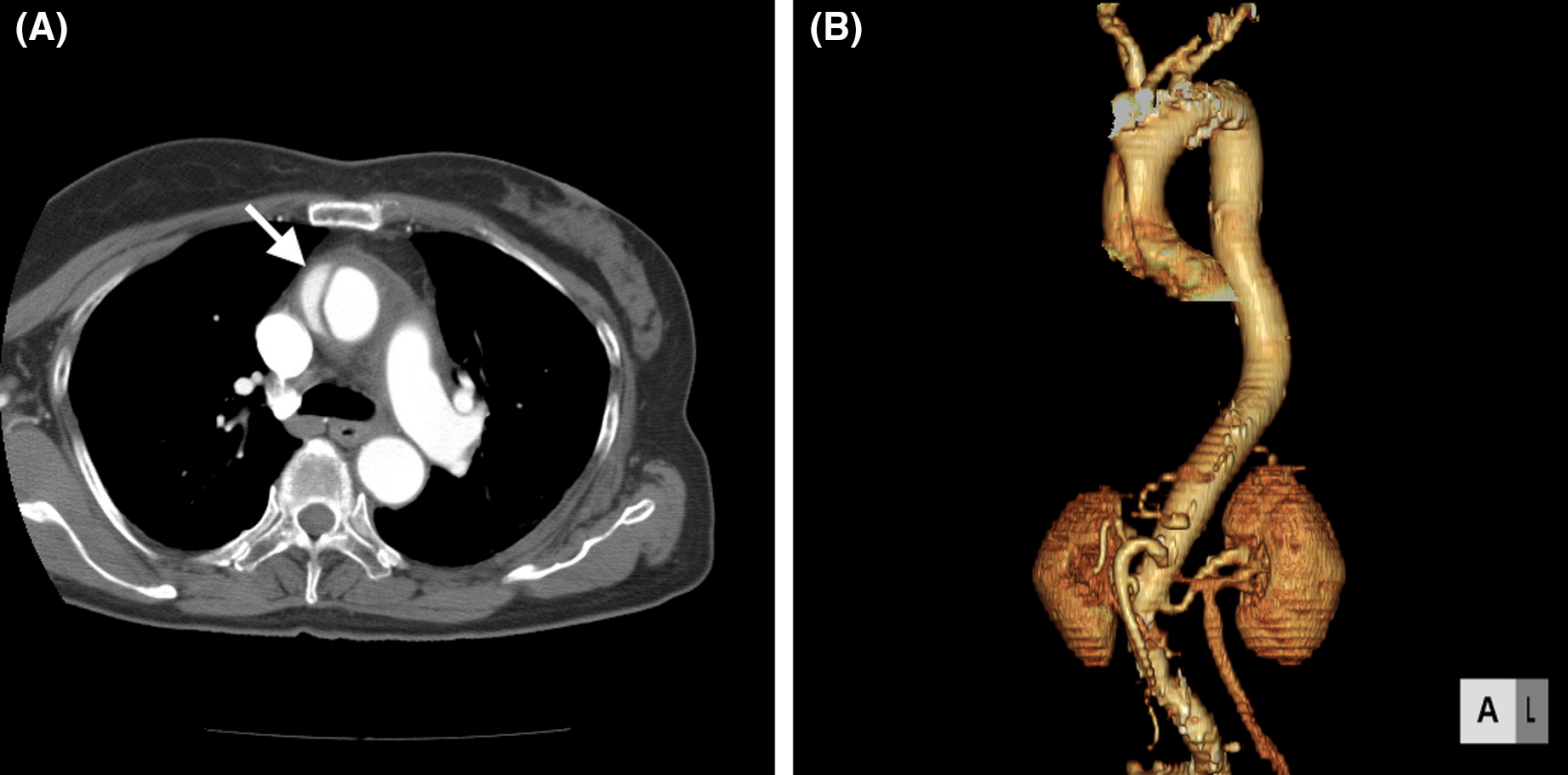
(A) Computed tomography imaging showing ascending aortic dissection with intimal flap (arrow). (B) 3D computed tomography imaging showing ascending aortic dissection.

**FIGURE 2:**
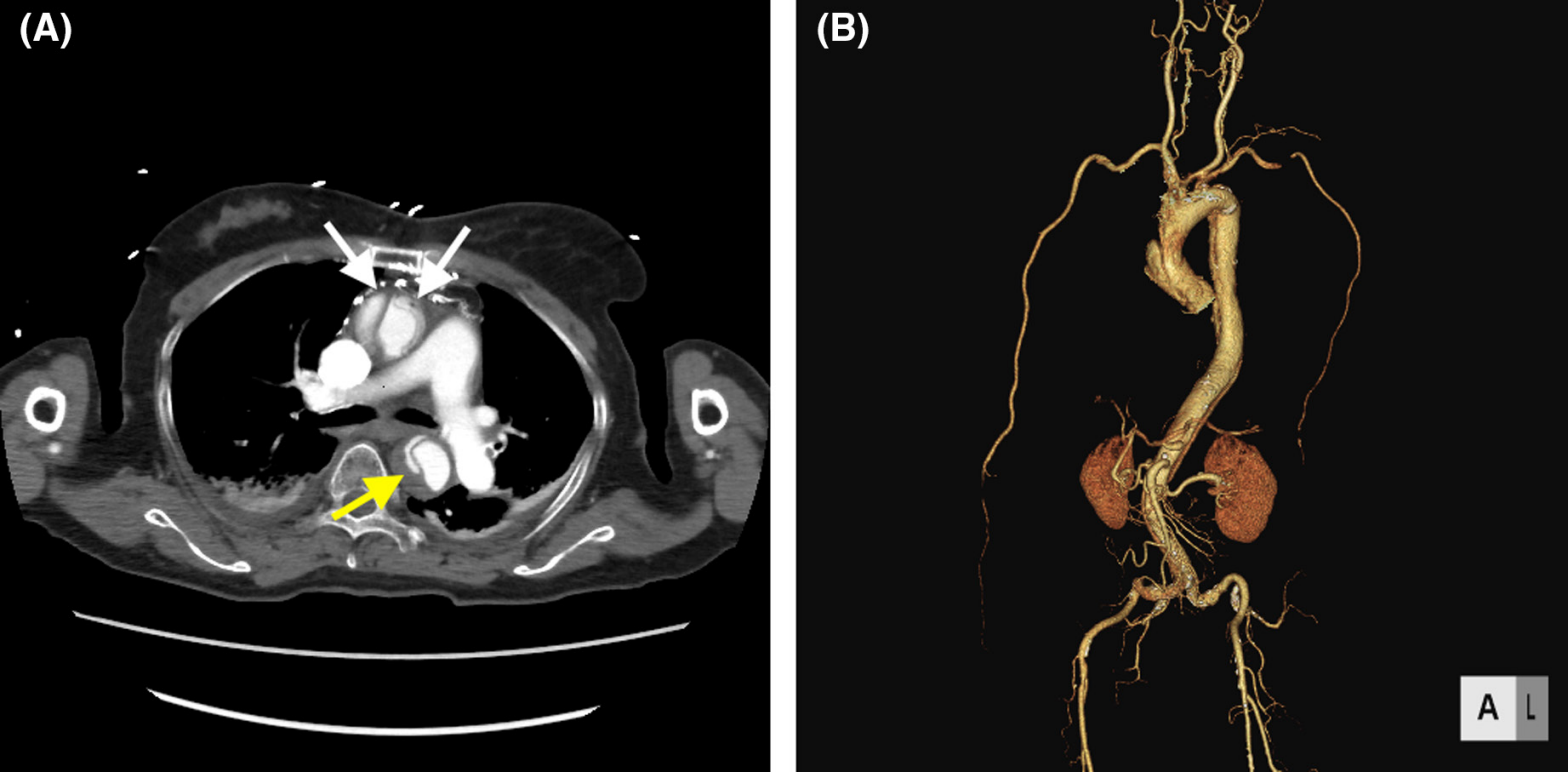
(A) Computed tomography imaging showing the new intimal flaps at ascending aorta (white arrow) and descending aorta (yellow arrow). (B) 3D computed tomography imaging showing the dissection progressed from ascending aorta to the terminal aorta.

**FIGURE 3:**
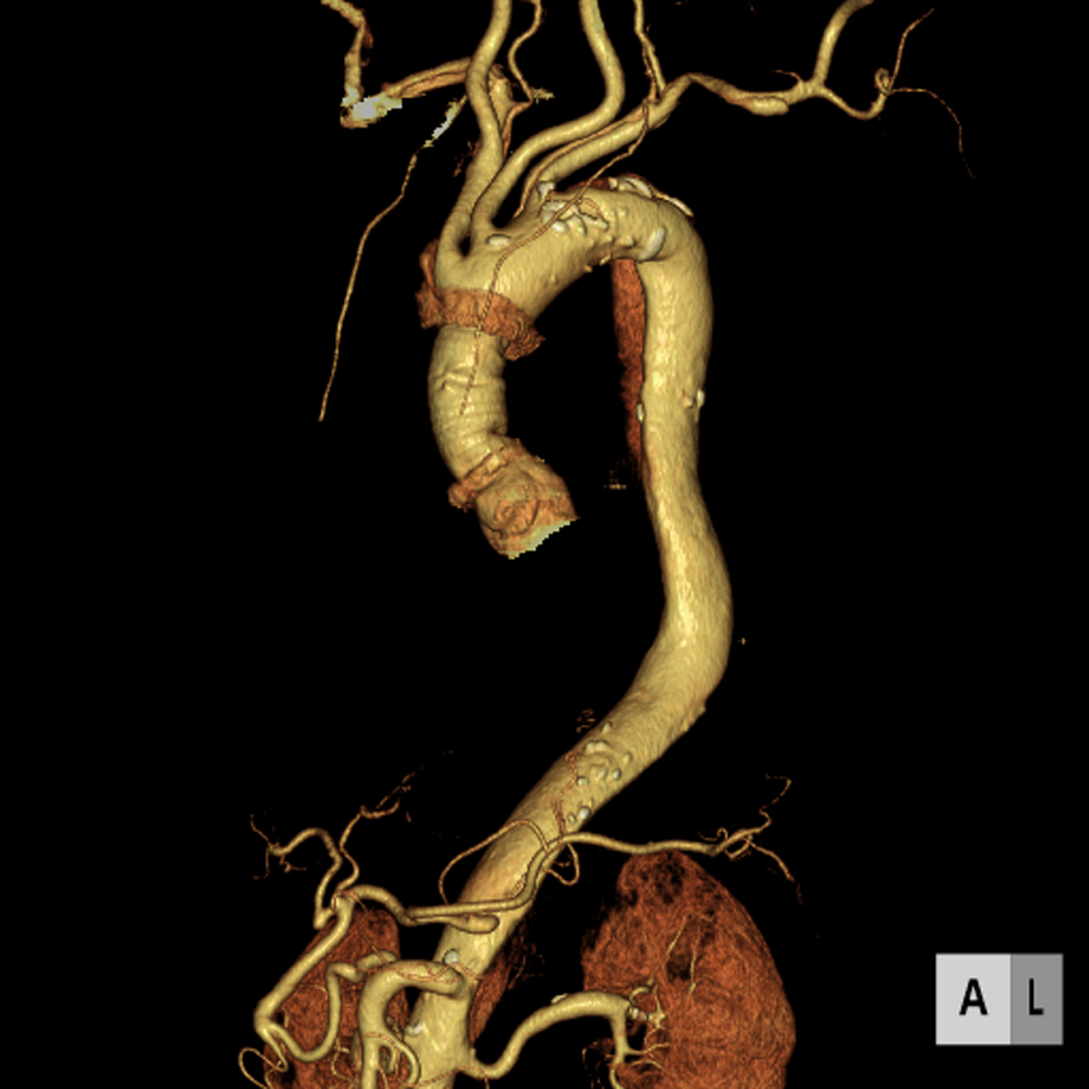
3D computed tomography imaging showing ascending aorta replaced with an artificial graft.

An 85‐year‐old woman was admitted to our hospital with a high‐energy motor vehicle trauma. The computed tomography (CT) imaging showed Stanford type A acute aortic dissection (AAAD) (DeBakey Type II), right clavicle fracture, and left first rib fracture. (Figure 1) The abdomen was bruised, and seatbelt trauma was suspected. To reduce the risk of hemorrhagic complication with use of heparin and cardiopulmonary bypass (CPB), the patient was scheduled for ascending aortic replacement after several days of strict blood pressure control rather than immediate emergency surgery. However, on the second day after admission, the patient developed a progression of the AAAD extending from the ascending to the descending aorta. (Figure 2) An emergent ascending aortic replacement was performed using Triplex 1 Branch graft (Terumo). (Figure 3) The brachiocephalic artery, the left common carotid artery, and the left subclavian artery were not reconstructed. Intraoperatively, the usual dose of heparin was used during CPB. The patient was hemodynamically stable, and transesophageal echocardiography and abdominal ultrasound confirmed there was no bleeding into the thoracic or abdominal cavities. No subcutaneous bleeding was observed. The usual dose of protamine was used to reverse heparin. No additional transfusion was required compared to ascending aortic replacement for AAAD. The patient had no significant complications, including stroke, and the postoperative course was uneventful. Given the advanced age of 85 and the limited availability of family assistance after discharge, the patient was transferred to a rehabilitation hospital where further rehabilitation.

Traumatic AAAD is rare, and often complicated with other hemorrhagic organ injuries, making it sometimes difficult to determine the timing of aortic repair. The first choice for AAAD is aortic replacement using CPB, and there is no effective endovascular device for AAAD with entry in the ascending aorta. The use of heparin and CPB can exacerbate hemorrhagic complications. Tsukioka and his colleagues have reported a successful standby total arch replacement for traumatic retrograde type A aortic dissection (RTAD) accompanied by intraperitoneal bleeding after 7 days of strict blood pressure control.^1^ However, the present case is different because it is not a RTAD, but antegrade dissection with an entry in the ascending aorta. Kim and his colleagues reported that the early mortality rate with medical therapy for antegrade dissection was 58.5%, significantly higher than that with medical therapy for RTAD (6.1%).^2^ Ito and his colleagues have reported successful emergent ascending aortic replacement for traumatic antegrade AAAD with sternum fracture.^3^ Although the risk of hemorrhagic complications must be evaluated in each case, the emergent aortic repair is required for traumatic antegrade AAAD rather than conservative therapy.

## AUTHOR CONTRIBUTIONS

**Hironobu Nishiori:** Conceptualization; data curation; formal analysis; investigation; methodology; project administration; resources; validation; visualization; writing – original draft; writing – review and editing. **Hisanori Fujita:** Conceptualization; data curation; formal analysis; investigation; methodology; project administration; resources; software; supervision; validation; visualization; writing – review and editing. **Seiichi Yamaguchi:** Conceptualization; data curation; formal analysis; investigation; methodology; project administration; resources; supervision; validation; visualization; writing – review and editing.

## FUNDING INFORMATION

None.

## CONFLICT OF INTEREST STATEMENT

None.

## ETHICS STATEMENT

None.

## CONSENT

Written informed consent was obtained from the patient to publish this report in accordance with the journal's patient consent policy.

## Data Availability

None.
